# Comparative genomics of Shiga toxin encoding bacteriophages

**DOI:** 10.1186/1471-2164-13-311

**Published:** 2012-07-16

**Authors:** Darren L Smith, David J Rooks, Paul CM Fogg, Alistair C Darby, Nick R Thomson, Alan J McCarthy, Heather E Allison

**Affiliations:** 1 Microbiology Research Group, Institute of Integrative Biology, University of Liverpool, Crown Street, Liverpool, L69 7ZB, UK; 2 Pathogen Genomics, The Wellcome Trust Sanger Institute, Wellcome Trust Genome Campus, Hinxton, Cambridge, CB10 1SA, UK

## Abstract

**Background:**

Stx bacteriophages are responsible for driving the dissemination of Stx toxin genes (*stx*) across their bacterial host range. Lysogens carrying Stx phages can cause severe, life-threatening disease and Stx toxin is an integral virulence factor. The Stx-bacteriophage vB_EcoP-24_B_, commonly referred to as Ф24_B,_ is capable of multiply infecting a single bacterial host cell at a high frequency, with secondary infection increasing the rate at which subsequent bacteriophage infections can occur. This is biologically unusual, therefore determining the genomic content and context of Ф24_B_ compared to other lambdoid Stx phages is important to understanding the factors controlling this phenomenon and determining whether they occur in other Stx phages.

**Results:**

The genome of the Stx2 encoding phage, Ф24_B_ was sequenced and annotated. The genomic organisation and general features are similar to other sequenced Stx bacteriophages induced from Enterohaemorrhagic *Escherichia coli* (EHEC), however Ф24_B_ possesses significant regions of heterogeneity, with implications for phage biology and behaviour*.* The Ф24_B_ genome was compared to other sequenced Stx phages and the archetypal lambdoid phage, lambda, using the Circos genome comparison tool and a PCR-based multi-loci comparison system.

**Conclusions:**

The data support the hypothesis that Stx phages are mosaic, and recombination events between the host, phages and their remnants within the same infected bacterial cell will continue to drive the evolution of Stx phage variants and the subsequent dissemination of shigatoxigenic potential.

## Background

Shiga toxin encoding bacteriophages (Stx phages) are responsible for converting the pathogenic profiles of their bacterial hosts. Enterohaemorrhagic *Escherichia coli* (EHEC), a subset of the Shigatoxigenic *E. coli* (STEC), differentiated by their ability to produce attachment and effacement lesions, emerged as a serious food borne threat to humans in the 1980s [1-3]. The emergence of this group of organisms was due to an Stx phage infection of a mildly pathogenic progenitor strain [4]. The severe disease (bloody diarrhoea and haemorrhagic colitis) and disease sequelae (haemolytic uraemic syndrome [HUS] and thrombotic thrombocytopenic purpura [TTP]) caused by EHEC are all linked to the activity of the Shiga toxin (Stx) [5], the expression of which is genetically coordinated by the lytic replication cycle of Stx phage [6]. Although the global incidence of EHEC infection is low, severe disease and death occurs in an unacceptably high proportion of infected individuals [7]: 10% and 3–5%, respectively [8].

Stx phages are lambdoid bacteriophages, sharing the distinct genome organisation of the archetypal bacteriophage lambda (λ) [5]. They possess two replication strategies: lysogenic, where the phage genome directs its integration into the bacterial host genome as a prophage; or lytic, where viral progeny are assembled intracellularly and released by lysis of the host cell through the action of phage encoded lysozyme, holin and pinholin proteins [1,9,10]. Production of Stx in the lysogen is linked to the latter, and the release of Stx from the lysogen predominantly coincides with induction of the lytic cycle and bacterial host cell lysis [6].

Bacterial genome sequencing projects have highlighted the impact that temperate phages have upon bacterial evolution, and those that impact directly on the pathogenicity of the host bacterium are known as converting phage. In addition to *stx* genes carried by Stx phages and expressed by *E. coli*, other examples of converting phage include the CTX phage encoding the cholera toxin genes expressed by *Vibrio cholerae*[11] and *lom* and *bor* of bacteriophage lambda, which affect *E. coli* adherence to human buccal epithelial cells [12] and sensitivity to serum killing [13], respectively. It can be postulated that the maintenance of converting phage in a lysogen is due to positive selection pressure for prophage carriage by the host cell in an animal host.

The bacteriophage vB_EcoP-24_B_[14], carrying the Shiga toxin 2 variant (Stx2) [5] (hereafter referred to as φ24_B_) has been well characterised [15-21] since its initial purification following induction from a clinical isolate of *E. coli* O157:H7 [22]. φ24_B_ infects rough and smooth strains of *E. coli*[18] and can adsorb to many members of the *Enterobacteriaceae,* including *Salmonella* spp [18]. The adsorption target for this phage is an essential outer membrane protein, BamA, which is involved in the biogenesis of the Gram negative bacterial outer membrane and is not only highly conserved across members of the *Enterobacteriaceae*, but also conserved to some degree in all Gram negative bacteria [20]. Using a Stx phage multi loci gene typing system [21], it was demonstrated that >70% of Stx phages share a gene responsible for the short-tailed phage morphotype that enables adsorption to BamA [20]. φ24_B_ also has the ability to multiply infect a single host cell and integrate into different sites across the *E.coli* chromosome [16,17,22], a behaviour which departs from the lambda phage immunity dogma [15]. This could act to not only increase the pathogenic profile of the host with each subsequent infection [23], but also enable recombination events between resident inducible and cryptic prophages, promoting the production and release of novel recombinant phage mosaics.

The objectives of this study were to sequence the genome of φ24_B_ and apply comparative genomic analyses to highlight important genetic similarities and differences across the Stx phages sequenced to date. The ultimate aim is to identify potential effectors controlling the biology of these phages and the expression of genes that provide a selective advantage to either the bacterial lysogen or to the phages themselves.

## Results and discussion

### Genome annotation

**Figure 1:**
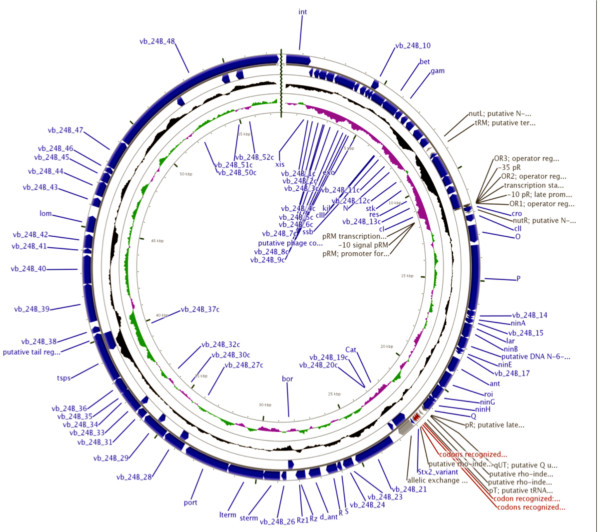
**CGView-derived schematic of the Ф24**_**B**_**genome; the concentric rings include the annotation, location and direction of expression.** Genes that are detailed in the centre of the genome and suffixed with a ‘c’ are expressed from the complimentary strand. The internal concentric rings indicate +/- GC skew and GC content.

**Figure 2:**
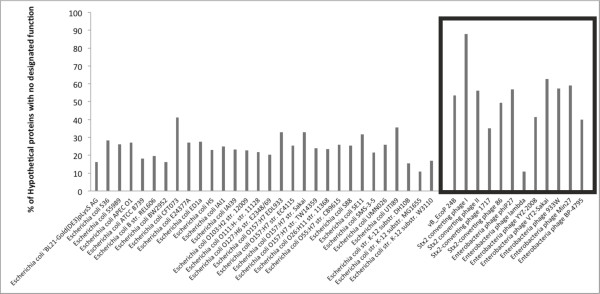
**Percentage levels of unknown/hypothetical genes with no inferred function from all sequenced and annotated*E. coli*strains and Stx phages available on Genbank.** The open box encompasses the current levels of annotated genes with no inferred function in Stx phage genomes.

Phage genes are usually small in size (< 1 kb), and very few of them have been subjected to detailed biochemical/functional characterisation, which makes the definitive annotation of phage genomes challenging. Notwithstanding the difficulties inherent in the production of informative phage genome annotation, the sequencing and subsequent annotation of the φ24_B_ genome is reported here [HM208303]. Its genomic organisation confirms that φ24_B_ is a lambdoid phage sharing similar overall genetic context with bacteriophage lambda (Figure 1). Annotation of the 57,677 bp genome revealed 88 putative coding regions (CDS, including the *stx*_*2*_*AB* genes of which the B gene is not annotated due to allelic replacement with the chloramphenicol resistance gene from pLysS [Novagen]), comprised of 26 CDS (30%) that shared a high level of sequence similarity with those of known function in other lambdoid phages (with or without *stx* genes); three CDS (vb_24B 2c, 4c and 25c), which have never been identified previously; eleven CDS sharing some, but not complete, homology to those genes with poorly defined roles in lambdoid phage biology; and 48 CDS encoding proteins of unknown function (55%), but are found in association with other lambdoid phages ( Additional file 1). A comparison of the number of genes encoding proteins of undetermined function in Stx phages and the number of hypothetical proteins encoded by sequenced *E. coli* isolates (Figure 2), demonstrates that Stx phages carry a greater percentage of hypothetical genes than their *E. coli* hosts, 55% verses 24%, respectively (Figure 2), especially remarkable considering the size differential between the bacteriophage and bacterial genomes, but not an uncommon occurrence in bacteriophage genomes [24]. An analysis of the annotated Ф24_B_ genome with CGView [25,26] (Figure 1) shows that hypothetical genes are particularly common in the late gene region of the phage; downstream of the antiterminator *Q*, 44 φ24_B_ genes were annotated (both strands) of which 32 (73%) are designated as hypothetical. Because of their location in the late gene region, their expression is likely to be linked to prophage induction/phage replication unless they are morons (horizontally acquired genes with no function for the phage, but usually beneficial to the bacterial host), uncoupled from the standard regulatory networks [27]. Expression analyses of these 32 genes is necessary to determine if they have been carried along via *in situ* recombination events without impacting the bacterial host or phage replication machinery or if they have been retained in the genome under their own expression control (or linked to other regulatory networks) because they benefit the bacterial host or phage replication.

Two unexpectedly large genes were identified in the Ф24_B_ genome sequence. The first of these large genes, vb_24B 48, is predicted to encode a protein of 2,808 aa and is located close to the right end of the genome (Figure 1). This gene is also carried by other Stx phages including 933W, VT2-Sa, Stx2 II, Stx2 converting bacteriophage 86 and Min27. Gene vb_24B 48 homologues have also been identified within bacterial genomes carrying non-Stx prophages, *e.g. Salmonella enterica* subsp. *enterica* serovar Kentucky isolate (ZP_0258689) encodes a gene sharing 1128 of the 1611 amino acid residues. The predicted protein of vb_24B 48 has no easily assignable function, but does possess a partial COG1483 domain (associated with the AAA + superfamily of ATPases by general function prediction) between residues 345 and 1176; SignalP analysis [28] indicates that the first 15 nucleotides might function as a leader peptide. The protein encoded by vb_24B 48 has no homology with any protein subjected to conventional functional analysis, but TMPred [29,30] predicts that the protein possesses membrane-spanning domains. This protein has many of the characteristics of the giant genes that typically encode surface proteins involved in bacterial fitness [31], and this could be relevant to its conservation among Stx phages. The second large gene, P (2906 bp), encodes the polymerase for Ф24_B_ replication. P_Ф24B_ possesses a number of well characterised and conserved domains, including an intact TOPRIM_primase domain (cd01029) at the amino terminus and an intact P loop NTPase superfamily domain (cl09099) at the carboxyl terminus, specifically harbouring the GP4d_helicase domain (cd01122). An orthologue of P_Ф24B_ has been found in association with a *Shigella flexneri* prophage (YP_690085.1, sharing 955 of the 968 amino acid residues), and in Stx phage Min27 (YP_001648921.1) with an amino acid identity of 87%. P_Ф24B_ carries an intein [32,33], interrupting amino acid residues 372–702, and includes an intact HintN domain (cl12032), specifically of the smart00306 superfamily. Comparison of the CDS sequence excluding the intein shows that P_Ф24B_ shares significant identity with a number of prophage or bacteriophage encoded proteins including ZP_0795005.1 and ZP_04535347.1 associated with an unclassified member of the *Enterobacteriaceae* (NZ_ADCU00000000), and an unidentified *Escherichia* isolate (NZ_DS999462.1), respectively. It is very likely that the activation of the intein will play a role in the post-translational regulation of the replication protein, but this, as well as the basic function of the intein, has yet to be experimentally determined.

φ24_B_ also harbours the two accessory genes, *lom* and *bor*[5], that in bacteriophage lambda are not involved in phage replication, but do affect the fitness of lambda lysogens in mammalian hosts [13,34] and are expected to play similar fitness roles in this Stx phage and other Stx phages that carry these genes.

### Genome comparisons

**Figure 3:**
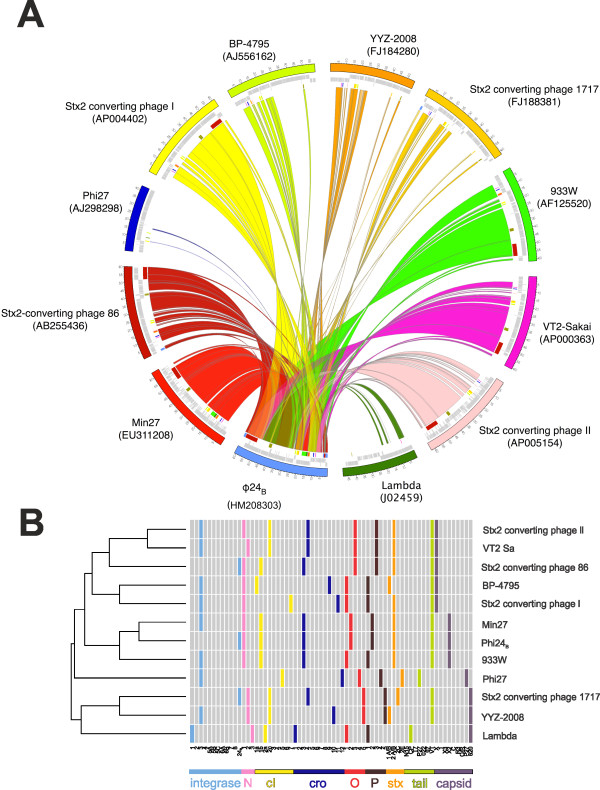
**Multi-genome comparison of all sequenced Stx phages, the archetypal lambdoid phage, Lambda and Ф24**_**B**_**. A.** Circos map depicting the MuMer alignment results with respect to Stx2 phage Ф24_B._ Each coloured segment represents a phage genome with the numbers on the external surface indicating genome size in kb. Inside the genome ring are hatch marks indicating gene locations and their respective coding strands. The inner circle is composed of coloured blocks that are indicative of gene conservation with Ф24_B_. The coloured swept arcs indicate sequence conservation and orientation of those sequences with respect to Ф24_B_. **B.** A multi-loci comparison [21]. Loci corresponding to the genome annotation that have been marked are loci that have been used in previous multi-loci typing of Stx bacteriophages or are defined in Additional file 1: Table S 1.

Ф24_B_ was compared to eleven previously sequenced Stx phages [35-43] (though Stx2 bacteriophage 86 ([AB255436] is unpublished) and bacteriophage lambda [39]. The analysis presented in Figure 3 highlights the mosaic nature of these lambdoid phages. The most similar Stx phages to Ф24_B_ are Min27 [36], 933W [41], VT2-Sakai [42], and the Stx2 converting phages 1 and 2 [35], which like Ф24_B_ all possess a *Podoviridae*-like morphology. These phages represent a global collection of Stx phages associated with incidents of human STEC infection from around the world e.g. 933W (US), Sakai and all Stx2 converting phages I, II and 86 (Japan), Stx 2 converting phage 1717 (Canada), Ф24_B_ (UK) and Min27 (China). These phages all share regions of homology with one another, but the degree of shared identity differs between phages, and no two phage are identical.

There is evidence that most circulating Stx phages are short-tailed *Podoviridae*[5,20,21,44,45], which have evolved an almost perfect infection strategy utilising an essential, highly conserved, outer membrane protein BamA (previously YaeT) for host cell recognition and adsorption [20]. This essential adsorption target, the fact that many outbreak strains carry more than one Stx phage [46,47], and the capacity of at least some Stx phages to multiply infect a single host cell [15-17,22,48] is likely to foster many opportunities to drive phage evolution through *in situ* recombination events. Thus the similarities in genome content across the short-tailed phages depicted in Figure 3, excluding lambda and Phi 27 that lie outside this group, may be a consequence of such recombination [49].

Genomic comparison has also shown that although many of the genes carried by Stx phages encode hypothetical proteins, there are recognisable accessory genes with activities that have been characterised in other systems, *e.g*. *exogambetlarlombor* and *stk*. The genes *exo, gam* and *bet* are the three components of the lambda-encoded Red recombinase system [50]. The products of these genes increase DNA recombination rates, which is likely to drive the creation of novel phages and extend bacterial host ranges through *in situ* recombination events between resident inducible and cryptic prophages, as well as infecting phages in the bacterial lysogen [5]. The gene *lar* encodes a protein involved in the alleviation of restriction systems [51], which are often used by bacteria as a primary defense against phage infection [52]. The genes *lom and bor* encode products that enhance the lysogen’s ability to colonise its host [13,34], and *stk* encodes a kinase with an as yet unidentified impact on the lysogen or the lysogen’s host [53], but it is clear that stk expression is controlled by the pRM promoter, and its expression occurs only under conditions of stable lysogeny [54].

The genes associated with the genetic switch, controlling the behaviour of these phages and their decision to enter the lysogenic or lytic replicative cycles (*e.g. cI, Q* and *N*), are present across all lambdoid phages, though distinct sequence variants are known (Figure 3). A PCR-based multilocus characterisation system developed for Stx phages [21] was applied to the 11 sequenced Stx phages and lambda (Figure 3B). The integrase gene of Ф24_B_[16,17] is also carried by the Stx2 converting phages 86 and 1717. All three phages possess the *int* genes in a genomic orientation opposite to the lambda phage integrase gene. The Ф24_B_-like integrase gene is under the control of its own promoter region [55] in all three phages from where it is likely to drive high frequency superinfection events [17]. The Ф24_B_*cIII* gene is not present in P27, but in the other phages it is well conserved sharing at least 99% aa identity. The antiterminator, N, involved in early gene expression, is present in one of three forms in all but phage P27. N1 [21] is present in Ф24_B_, 933W, Stx2 converting phage I, Min27 and BP 4795, all sharing at least 98% identity, and N2 [21] is carried by VT2Sa, Stx2 phage 1717 and YYZ-2008, whilst Lambda possesses a third variant ( Additional file 1: Table S1)_._; the three variants can share as little as 22% sequence identity. The *cI* gene product, the regulator controlling maintenance of lysogeny through repression of the lytic life cycle, was identified in five variant forms. The repressor of Stx phages 933W, Min27, Stx2 converting I and Stx2 converting phage 86 all possess *cI*_*1b,*_ while BP-4795 possesses *cI*_*1a*_, which shares 69% overall identify with the cI1a protein and 100% identity at the carboxy terminal half. Sequence and structure/function predictions mean that the altered amino terminus is likely to have different DNA binding properties, whilst retaining similar dimerization properties that are key to its function [56]. The *cI*_*2c*_ genes from Vt2-Sa, Stx2 phage II, YYZ-2008 and Stx2 phage 1717 all share sequence identity across the entire coding region of the *cI* gene, though they are currently annotated with different amino termini. The VT2-Sa *cI* gene amplifies with the *cI*_*2c*_ primers, but a single nucleotide polymorphism has introduced a stop codon and thus ablates 60 amino acids from the amino terminus, probably destroying the ability of this repressor protein to bind DNA; this may, at least partly, explain the non-inducible nature of this prophage [57]. The archetypal Lambda repressor (CI_2a_) shares 100% identity at its carboxy terminus with the CI_2c_ variants, but its amino terminal end is unique, and again implies that it binds DNA differently from the CI_2c_ variants. The Stx2 converting phage I possesses the *cI*_*7*_ variant ( Additional file 1: Table S1) not previously included in the Stx phage multilocus PCR typing system [21]. Orthologues of the *cro* gene product (Cro_3_) are carried by Stx phages 933W, Stx2 converting phage 86, Ф24_B_ and Min27 and are all identical at the aa level. The *cro* gene variant (*cro*_*4*_) is carried by Stx2 converting phages 1717 and II as well as VT2-Sa, again sharing 100% amino acid identity. Lambda phage encodes Cro_1_; BP4795, Cro_9_; YYZ2008, Cro_10_; Stx2 converting phage I, Cro_11_ and P27, Cro_12_. All the diversity seen across the *cI* variants and the lack of association of specific *cI* genes with specific *cro* genes (Figure 3B) has been predicted [58], providing evidence of repressor/operator coevolution. This coevolution has been predicted to drive superinfection immunity groups and thus effect the production of new and novel Stx phage mosaics [5]. Only the CII from Min27 is completely identical to that of Ф24B; all the other phages in the Circos comparison, apart from P27 and Lambda, have CII proteins that are approximately 86% identical at the protein level. Lambda CII has the lowest sequence identity at 36% and no orthologue was identified in P27.

Only Stx phage Min27 carries *O* and *P* genes (O_2_ P_2_; Additional file 1: Table S1) like those carried by Ф24_B_ (99 and 98% identity, respectively). Across all of the phages, there were five distinct DNA replication systems encoded, with little homology shared between each system. O_1_/P_1_ is carried by Lambda phage, 933W, Stx2 converting phage I and BP-4795; O_3_/P_3_ is carried by Stx2 converting phage II, VT2-Sa and Stx2 converting phage 86, O_4_/P_4_ is carried by P27 and O_5_/P_5_ is carried by Stx2 converting phage 1717 (Figure 3B). These two-protein systems would therefore be a suitable additional diversity marker for phage characterisation (Additional file 1: Table S1). The lytic induction enhancer, *Ant*, [55] can also be identified in genomic context within the genomes of Min27 (97%), VT2Sa and Stx2 converting phage II (78%) and Stx2 converting phage 1717 (73%) (Figure 3A). Downstream of *Ant* is a gene encoding a protein of similar predicted conformation, Roi, which shares its 125 amino-terminal amino acid sequence (242 a.a. in total) with Roi from bacteriophage HK022 [59]. In bacteriophage HK022, Roi has been implicated in phage lytic growth [59]. Roi_Ф24B_ is identical at the protein level to Roi_Min27_, and possesses 99% sequence identity to the Roi genes of five of the other Stx phages. Roi encoded by genes carried by Stx2 converting phage II and VT2Sa, and Stx converting bacteriophage 86 are still distinctly similar but share lower identity to Roi_Ф24B_ (89 and 83%, respectively); in all cases the genomic context of Roi in these Stx phages is preserved. The protein product of the antiterminator gene *Q* is widely conserved (≥98% identity) throughout the Stx phages, as it is in all lambdoid phages [60]. The well conserved short tail of Ф24_B_ is widespread across Stx phages [21] due to its outer membrane protein adsorption target that is itself highly conserved and an essential gene in the bacterial host [20]. Examination of the distribution and similarity of the gene encoding this short tail structure across the sequenced Stx phages, 933W, VT2Sa, Min27, Stx2 converting phage 2 and Ф24_B_ reveals 99% sequence identity at the protein level. This 1% difference is simply due to different start codons. Stx2 converting phage 1 possesses a tail gene with 95% identity to Ф24_B_.

A Jaccard dissimilarity dendrogram (Figure 3B) was created from data on the presence or absence of the gene variants associated with each sequenced genome. The dendrogram illustrates the high level of genetic diversity that exists amongst these 11 Stx phages, with no two phage possessing an identical genetic profile. This further demonstrates the genetic heterogeneity of Stx phages previously revealed by PCR multilocus typing of phage pools induced from STEC strains (55).

The most challenging question in phage genomics is: What is the function of the uncharacterised genes that dominate bacteriophage genomes? Phage genomes are normally small and compact, and it is likely that many of the genes of unknown function have been maintained in this dynamic pool by positive selection pressure. Most Stx phages have larger genomes than bacteriophage lambda, so carry more genes that are not required for core lambdoid phage replication and life cycle control. The suggestion that these accessory genes have roles in the fitness of either the Stx phages themselves or their bacterial hosts can be made with some confidence.

## Conclusions

Over the last 10 years, the phage research community has begun to use genomic analyses to compare double stranded DNA phages, most extensively with respect to the comparative genomics of mycobacteriophages or their lysogens [61-69]. Bacteriophages are significant drivers of bacterial evolution because of their ability to disseminate DNA across their host range, either as converting phages [70] or through both generalised (59) and specialised (25) transduction. By identifying genetic variation in groups of phage which impact upon the phenotypic profiles of their hosts, it may be possible to infer biological roles for the numerous hypothetical proteins identified in translated bacteriophage genome sequences.

In this full genomic comparison of eleven Stx phages we have demonstrated that no two sequenced Stx phage are identical. All of the lambdoid phages are mosaics, sharing genomic loci and genomic synteny, but to varying degrees. The short-tailed Stx phages possess more genomic relatedness, which may be driven by their shared host range (due to the adsorption target, BamA) enabling appreciable levels of genomic recombination, facilitating efficient recombination of and selection for genetic material carried by these phages. The phage backbone of P27 is very different from the other Stx phages and may be the result of a productive recombination even between a non-lambdoid and a lambdoid phage, as many key regulatory lambdoid phage elements cannot be identified within the P27 genome. However, the Shiga toxin genes remain linked to the *Q* gene. It has been reported before that lambdoid phages appear to possess most genetic morons within the late gene region [27], and the Stx phages hold true to this observation. The conserved nature of many of these morons, which are likely to confer some as yet unidentified property to their host cell, indicate that Stx phages are likely to contribute more to their pathogenic bacterial host than toxin production. Understanding these factors is likely to be important to understanding the evolution of EHEC and other Shiga toxin producing enteric pathogens.

Genomic approaches to phage biology provide the means to examine the growing number of novel bacteriophages isolated directly from different environments, induced from their bacterial hosts or identified as prophages in sequenced bacterial genomes. Deep pyrosequencing technologies, enabling metaviral analyses of environmental samples, are further driving our understanding and appreciation of bacteriophage genomics and the bacteriophage pan-genome [71,72]. Assigning definitive or putative functions to the hypothetical proteins that are the expressed products of the majority of bacteriophage genes remains the main barrier to significant progress in unravelling bacteriophage biology.

## Methods

### Bacterial strains and bacteriophages

The *E. coli* C derivative strain WG5^rif+^ and the *E. coli* K12 strain DM1187 have been used to isolate and propagate a number of Stx phages previously [15,16,18,21,22,73]. Unless stated otherwise, these bacterial strains were grown in Luria-Bertani broth (VWR) or on plates prepared by addition of 1.5% (w/v) agar (Difco). The engineered variant of Ф24_B_ sequenced in this study, Ф24_B_::Cat [22], possesses a *stx* operon that has been replaced with the *cat* gene, which confers chloramphenicol resistance upon its lysogen.

### VB_ECOP-24_B_::Cat (Ф24_B_::Cat) DNA extraction for genome sequencing

Agar plates with semi-confluent plaques of Ф24_B_::Cat were flooded with 3 ml of SM buffer (50 mM Tris Cl [pH 7.5], 0.1 M NaCl, 10 mM MgSO_4_,) [74] and gently agitated overnight at 4°C. The SM buffer was harvested and the plate flooded again with SM buffer. The top agar containing the plaques and the second volume of SM buffer were then scraped from the agar plates and added to the former sample. This mixture was vortexed, and the top agar and bacterial debris pelleted by centrifugation (10,000 g, 10 min). Chloroform (30 μl 10 mL^-1^) was added to the recovered supernatant to inactivate any remaining bacterial cells. Contaminating bacterial DNA and RNA were removed by the addition of DNAse (Ambion; 5 μg mL^-1^) and RNAse (1 μg mL^-1^), and the mixtures were incubated at 37°C for 1 hr. The phages present were precipitated in the presence of 33% PEG 8000 (Sigma) on ice for 30 min and recovered by centrifugation at 10,000 g for 10 min. The resulting phage pellet was suspended in 500 μl of SM per 30 ml original vol followed by a further DNAse and RNAse digestion. The viral nucleic acid was purified following two extractions with an equal vol 25:24:1 phenol:chloroform:isoamyl alcohol and centrifugation (14,500 g, 30 min). The DNA present was precipitated by the addition of 0.6 vol isopropanol. The DNA was harvested by centrifugation (14,500 g for 30 min), washed with 70% ethanol and allowed to air dry. It was then suspended in 100 μl of distilled H_2_O [60].

### Ф24_B_::Cat Sequencing and annotation

The Ф24_B_::Cat phage genome was sequenced at the Welcome Trust Sanger Institute. The phage DNA was randomly sheared by sonication and a library produced by cloning fragments into the plasmid pUC19 (New England Biolabs). The phage genome was sequenced to provide 10x coverage using the ABI3730 sequencer (Applied Biosystems). Assembly of the sequence was accomplished using Phrap, and contiguous sequence was assembled using GAP4. The phage DNA predicted coding genes were identified using ORPHEUS28 and GLIMMER29 and these predictions were combined and annotated in Artemis [75] by comparison against the non redundant database using BLASTN and TBLASTX [76]. Putative coding sequences were added to the annotation if they contained both start and stop codons and a probable ribosome binding site.

### Genome comparison

The accession numbers for the Stx phages used for the genome comparison were: Ф24_B_::Cat (HM208303), 933W (AF125520), P27 (AJ298298), Min27 (EU311208), Stx2 Converting phage I (AP004402), Stx2 Converting phage II (AP005154), Stx2 Converting phage 86 (AB255436), Stx2 Converting phage 1717 (FJ188381), VT2-Sakai (AP000363) YYZ 2008 (FJ184280), BP-4795 (AJ556162) and non-Stx encoding bacteriophage Lambda (J02459). Comparative genome analysis was performed using MUMmer version 3 [77] and visualized using CIRCOs [78]. Coordinates were generated using NUCmer [77] with the parameters breaklen, maxgap, mincluster, and minmatch set to 200, 90, 65 and 20, respectively.

#### R-based loci comparisons

The presence of bands from each individual amplification reaction, using primer pairs specific for variant loci [21], was used as the data for construction of a binary similarity matrix. Computation script was written using R version 2.11.1, to enable visualisation of the variant of each genetic locus present.

## Competing interests

The authors declare that they have no competing interests.

## Authors’ contributions

All authors have been involved in the writing of this manuscript and have approved the final version. PCMF prepared the phage genomic DNA and NRT sequenced and assembled the genome. HEA and DLS annotated the genome and DLS and ACD undertook the comparative genome analyses. DJR performed and analysed the multi locus characterisation. AJM and HEA conceived and directed this research and the ensuing manuscript. DLS and DJR are joint first authors and HEA is the corresponding author.

## Supplementary Material

Additional file 1 Table S1.Suggested primer set additions for Stx phage characterisation. Click here for file
