# Supplementary material for: Comparative genomics of Shiga toxin encoding bacteriophages
Source: BMC Genomics. 2012 Jul 16;13:311. doi: 10.1186/1471-2164-13-311 (PMC3430580; doi:10.1186/1471-2164-13-311)
Supplement: Additional file 1 — Table S1.Suggested primer set additions for Stx phage characterisation. [file 1471-2164-13-311-S1.doc]

Supplementary Table 1. Suggested primer set additions for Stx phage characterisation.

| **Gene Variant** | **Direction** | **Primer Sequence** |
| --- | --- | --- |
| N_3_ | Fwd | 5’-ATGTGYCARAGYMGNGG-3’ |
|  | Rev | 5’-GGDATNSWYTTNCCYTTD-3’ |
| cI_7_ | Fwd | 5’-ATHYTNGGNGUNMGNGC-3’ |
|  | Rev | 5’-RGANCCNCKNACYTCNG-3’ |
| cro_10_ | Fwd | 5’-atggarmgnacnwsntay-3’ |
|  | Rev | 5’-RAAYTGCATRTCDATYTC-3’ |
| cro_11_ | Fwd | 5’-atgytnmgnacnwsta-3’ |
|  | Rev | 5’-raanardatrtaraadat-3’ |
| cro_12_ | Fwd | 5’-atgacnytntangarath-3’ |
|  | Rev | 3’-nacytcnswytgyttncc-3’ |
| O_1_ | Fwd | 5’-atgacnaayacngcnaarath-3’ |
|  | Rev | 5’-rtcnacnccrtadatccartc-3’ |
| O_2_ | Fwd | 5’-atgwsnatgaawytnatggc-3’ |
|  | Rev | 5’-ckrtcraaraanacrtancc-3’ |
| O_3_ | Fwd | 5’-atgwsnaayathwsnaayytngc-3’ |
|  | Rev | 5’-atgngcrctyttytgyctytt-3’ |
| O_4_ | Fwd | 5’-atgcgngarggncayggntay-3’ |
|  | Rev | 5’-gcngcnacnccnccdatrtg-3’ |
| O_5_ | Fwd | 5’-atgggngtngtnaarytngc-3’ |
|  | Rev | 5’-gcdatnccnckrtcngcraa-3’ |
| P_1_ | Fwd | 5’-atgaaraayathgcngcncar-3’ |
|  | Rev | 5’-nacnswngcnccyttnarncc-3’ |
| P_2_ | Fwd | 5’-atgacnccnwsngarytngay-3’ |
|  | Rev | 5’-rtaytcngtnacrttytcytg-3’ |
| P_3_ | Fwd | 5’-atgacngayaayttytaygc-3’ |
|  | Rev | 5’-raanccnccyttyttyttns-3’ |
| P_4_ | Fwd | 5’-atgacnacnccngtntggagr-3’ |
|  | Rev | 5’-raaraayctytccatyttns-3’ |
| P_5_ | Fwd | 5’-atgmgncargayathgargc-3’ |
|  | Rev | 5’-acrtcngcnccytgngcrta-3’ |
| 24_B_ intein | Fwd | 5’-garccnaathytnytngcng-3’ |
|  | Rev | 3’-gcrcartcnacrtayctncc-3’ |
